# Recombinant antibodies recognize conformation-dependent epitopes of the leucine zipper of misfolding-prone myocilin

**DOI:** 10.1016/j.jbc.2021.101067

**Published:** 2021-08-09

**Authors:** Athéna C. Patterson-Orazem, Ahlam N. Qerqez, Laura R. Azouz, Minh Thu Ma, Shannon E. Hill, Yemo Ku, Lisa A. Schildmeyer, Jennifer A. Maynard, Raquel L. Lieberman

**Affiliations:** 1 School of Chemistry & Biochemistry, Georgia Institute of Technology, Atlanta, Georgia, USA; 2 Department of Chemical Engineering, University of Texas at Austin, Austin, Texas, USA; 3 Department of Molecular Biosciences, University of Texas at Austin, Austin, Texas, USA

**Keywords:** antibody, conformational, myocilin, misfolding, glaucoma, IOP, intraocular pressure, LZ, leucine zipper, OLF, olfactomedin domain, SEC, size-exclusion chromatography, TM, trabecular meshwork

## Abstract

Recombinant antibodies with well-characterized epitopes and known conformational specificities are critical reagents to support robust interpretation and reproducibility of immunoassays across biomedical research. For myocilin, a protein prone to misfolding that is associated with glaucoma and an emerging player in other human diseases, currently available antibodies are unable to differentiate among the numerous disease-associated protein states. This fundamentally constrains efforts to understand the connection between myocilin structure, function, and disease. To address this concern, we used protein engineering methods to develop new recombinant antibodies that detect the N-terminal leucine zipper structural domain of myocilin and that are cross-reactive for human and mouse myocilin. After harvesting spleens from immunized mice and *in vitro* library panning, we identified two antibodies, 2A4 and 1G12. 2A4 specifically recognizes a folded epitope while 1G12 recognizes a range of conformations. We matured antibody 2A4 for improved biophysical properties, resulting in variant 2H2. In a human IgG1 format, 2A4, 1G12, and 2H2 immunoprecipitate full-length folded myocilin present in the spent media of human trabecular meshwork (TM) cells, and 2H2 can visualize myocilin in fixed human TM cells using fluorescence microscopy. These new antibodies should find broad application in glaucoma and other research across multiple species platforms.

Antibodies are indispensable and ubiquitous tools for targeting a specific antigen that are widely used across biomedical research fields. Advances in antibody engineering methodologies (1) and concerns over the reproducibility of antibody reagents (2) are fueling a movement toward highly characterized recombinant antibodies (3). Recombinant antibodies avoid the batch-to-batch variability of polyclonal antibodies (4) and the genetic drift of monoclonal antibodies (5), while also allowing for facile conversion to other formats (human, mouse, or rabbit constant regions, Fab fragments). Such antibodies can be designed with the structure, function, and dysfunction of their targets in mind and can be combined with constant domains that allow for use of the same reagent on samples from human and animal tissues. Conformationally selective antibodies can detect, for example, amyloid (6, 7) and other misfolded protein aggregates (8, 9), with uses ranging from structural and mechanistic characterization to therapeutic applications. Antibodies recognizing distinct conformations of misfolding-prone and amyloidogenic proteins can help characterize misfolding pathways, determine the contributions of specific isoforms to disease, and may lead to therapies that block steps in the misfolding pathway or target-specific isoforms for destruction (10, 11, 12).

Myocilin is an extracellular multidomain protein (Fig. 1, *A* and *B*) that is highly expressed throughout the human body (13). Myocilin is best known for its high expression in the trabecular meshwork (TM) (14, 15), an ocular tissue in the anterior segment involved in the filtration of aqueous humor and the regulation of intraocular pressure (IOP) (16). IOP is the causal risk factor for vision loss in glaucoma, a disease estimated to affect ∼65 million people worldwide in 2020 (17). Mutations in the myocilin olfactomedin domain (OLF, Fig. 1, *A* and *B*) (18, 19) that lead to aggregation and intracellular sequestration (20) result in hereditary primary open-angle glaucoma. Myocilin expression is increased upon other glaucoma-relevant stressors such as steroid treatment (15) and mechanical stretching (21), suggesting broad relevance of myocilin to the TM and ocular pressure homeostasis. Additional studies of myocilin in skeletal muscle suggest a role in hypertrophy (22, 23), and implicate myocilin in other human diseases (24, 25, 26) including of the liver, heart, and kidney. The explicit biological function of myocilin in TM and other tissues remains unknown but functional implications for myocilin, requiring its proteolysis to release the OLF domain (27) and redox-dependent disulfide bond shuffling that leads to its multimerization (28, 29), have been suggested.

**Figure 1:**
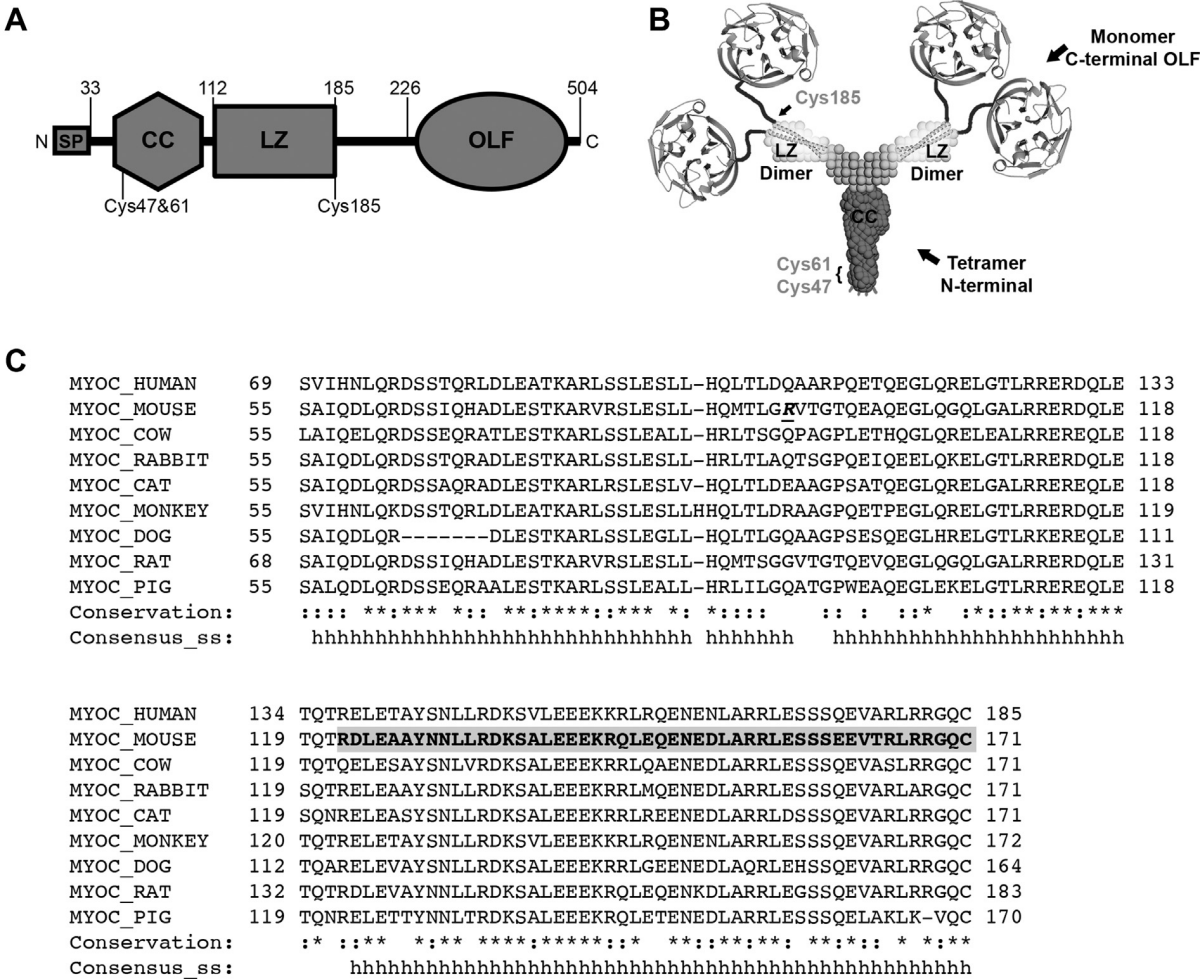
**Myocilin structure and sequences relevant to this study.***A*, myocilin gene encodes a signal peptide (SP), coiled-coil (CC), leucine-zipper (LZ), and C-terminal olfactomedin (OLF) domain. *B*, model of myocilin architecture based on SEC-SAXS, X-ray crystallography and chemical cross-linking (32). Reprinted with permission from Patterson-Orazem *et al.* (31). *C*, PROMALS3D (72) sequence alignment of myocilin from different animals used in glaucoma research. Accessions: Human, Q99972; Mouse, O70624; Cow, Q9XTA3; Rabbit, Q866N2; Cat, Q594P2; Monkey, Q863A3; Dog, Q2PT31; Rat, Q9R1J4; Pig, AAN59763. Conservation, :, similar; ∗, identical. Consensus_ss: h, helical secondary structure. ***R***, start of mLZ (Table 1), after inadvertent cleavage by Factor Xa. *Bold*, *gray shadow*, structurally characterized portion of mLZ (PDB code 5VR2) (32).

Myocilin antibodies are used routinely to track the protein in a variety of human samples and animal models, as well as to validate TM cell lines (30). Recently, we tested the available commercial myocilin antibodies to clarify the specific epitopes targeted (31). These antibodies recognized several epitopes across the myocilin protein, but were unable to distinguish between folded and misfolded forms. The ability to track different forms would help understand familial mutations that predispose the carrier to myocilin misfolding and heritable glaucoma. In addition, just one of four commercial antibodies (R&D Systems MAB3446) recognizes both human and mouse myocilin in western blot (unpublished observation). Thus, the antibodies used to study myocilin, including their inability to distinguish among conformational states, are likely contributing to the still-blurry picture of myocilin function.

Here, we leveraged our understanding of myocilin structure to develop the first antibodies with defined conformational selectivity for the leucine zipper (LZ) domain. We focused on antibodies binding N-terminal domains first (Fig. 1*B*) because constructs have been characterized that are not prone to aggregation (32), which limits the overall myocilin conformational heterogeneity. The top ranked antibodies bind the myocilin LZ domain from human and mouse (∼80% sequence identity, Fig. 1*C*) with low nanomolar affinity, which streamlines laboratory reagents and supports comparisons of animal and clinical samples. Sensitivity of the LZ conformational state, defined by comparing immunoreactivity in ELISA, dot blot, and western blot, identified one highly conformationally selective and one less selective antibody. Both antibodies immunoprecipitate full-length myocilin from spent media of primary human TM cells. Additional maturation steps applied to the conformationally selective antibody yielded a final antibody suitable for fluorescence imaging of myocilin in fixed human TM cells. These new antibodies should find broad application in glaucoma and other research areas where myocilin plays a role.

## Results

### Development and identification of candidate antibody sequences

**Figure 2:**
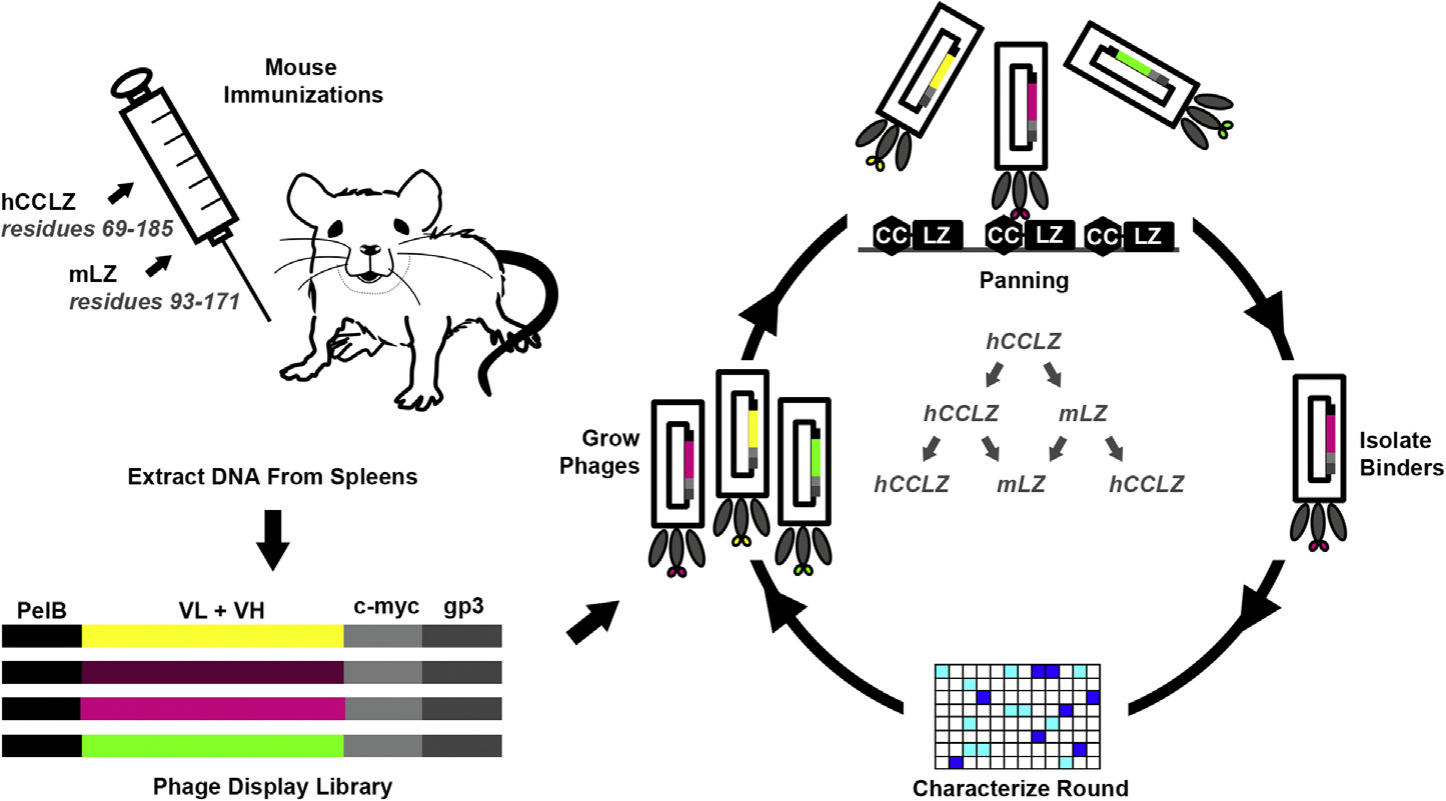
**Schematic workflow for mouse immunizations, phage display library construction, and *in vitro* selection of candidate antibody sequences.** Mice were immunized and boosted with alternate rounds of hCCLZ and mLZ. Antibody sequences were amplified from mouse spleens with degenerate primers and cloned to generate a phage display library, which was subjected to successive rounds of panning against both immunogens. After three rounds of panning, clones with strong expression and cross-reactive binding to hCCLZ and mLZ were identified by indirect ELISA and sequenced.

Mice were immunized (Fig. 2) with purified human CCLZ (hCCLZ; Table 1), a well-characterized and stable N-terminal structural domain comprising residues 69–185 that includes both dimer and tetramer regions (32). Mice were given boosters alternating between hCCLZ and mouse LZ (mLZ) until strong titers to both immunogens were detected in mouse sera (Fig. S1*A*). While our intent was to conduct booster immunizations with the mouse CCLZ homolog (mCCLZ; comprising residues 55–171, Table 1), we later learned that secondary cleavage by Factor Xa during purification resulted in a smaller mLZ immunogen (residues 93–171, Table 1) (32). Antibody DNA libraries were generated by extracting RNA from spleens of mice exhibiting immune responses and cloned into scFv format for phage display panning to generate a library comprised of 1.5 × 10^7^ individual clones. Sanger sequencing of ten individual colonies and *BstN*I fingerprinting of ∼20 clones were all unique, indicating the library included many diverse sequences (Fig. S1, *B* and *C*). The presence of scFvs with premature stop codons that therefore lack C-terminal c-myc tags was reduced by panning against an anti-c-myc antibody. Myocilin-targeting antibodies were then enriched in the next three successive rounds by panning against hCCLZ and mLZ (Fig. 2) before analysis as individual phage clones in ELISA and sequencing of 80 positive clones. In all, 13 unique clones were selected for further characterization based on (i) good expression, (ii) specific yet sensitive myocilin binding in a monoclonal phage ELISA, (iii) binding to human and mouse homologs, and (iv) diverse sequences for further characterization as a purified single-chain antibody (scAb; a single polypeptide composed of the variable heavy chain, variable light chain, and human constant kappa domain).

**Table 1:** Protein constructs used in this study

Construct	Description
hTM myocilin	Full-length myocilin from spent primary human TM cell culture
hNTD (33–185)	Recombinant human myocilin construct lacking OLF domain
hCCLZ (69–185)	V-shaped tetramer composed of part of human myocilin CC and all of LZ
hLZ (112–185)	Dimeric human myocilin LZ construct
hCC (33–111)	Tetrameric human myocilin CC-only myocilin construct
mCCLZ (55–171)	Equivalent construct to hCCLZ but from mouse myocilin
mLZ (93–171)	Truncated dimeric LZ from mouse, resulting from cleavage by Factor Xa
Control lysate	*E. coli* lysate expressing another protein with same tags as recombinant myocilin constructs

Residues in human or mouse myocilin that encompass the recombinant protein constructs are shown in parenthesis; previously reported in (32).

### Initial evaluation of LZ-targeting scAbs

**Figure 3:**
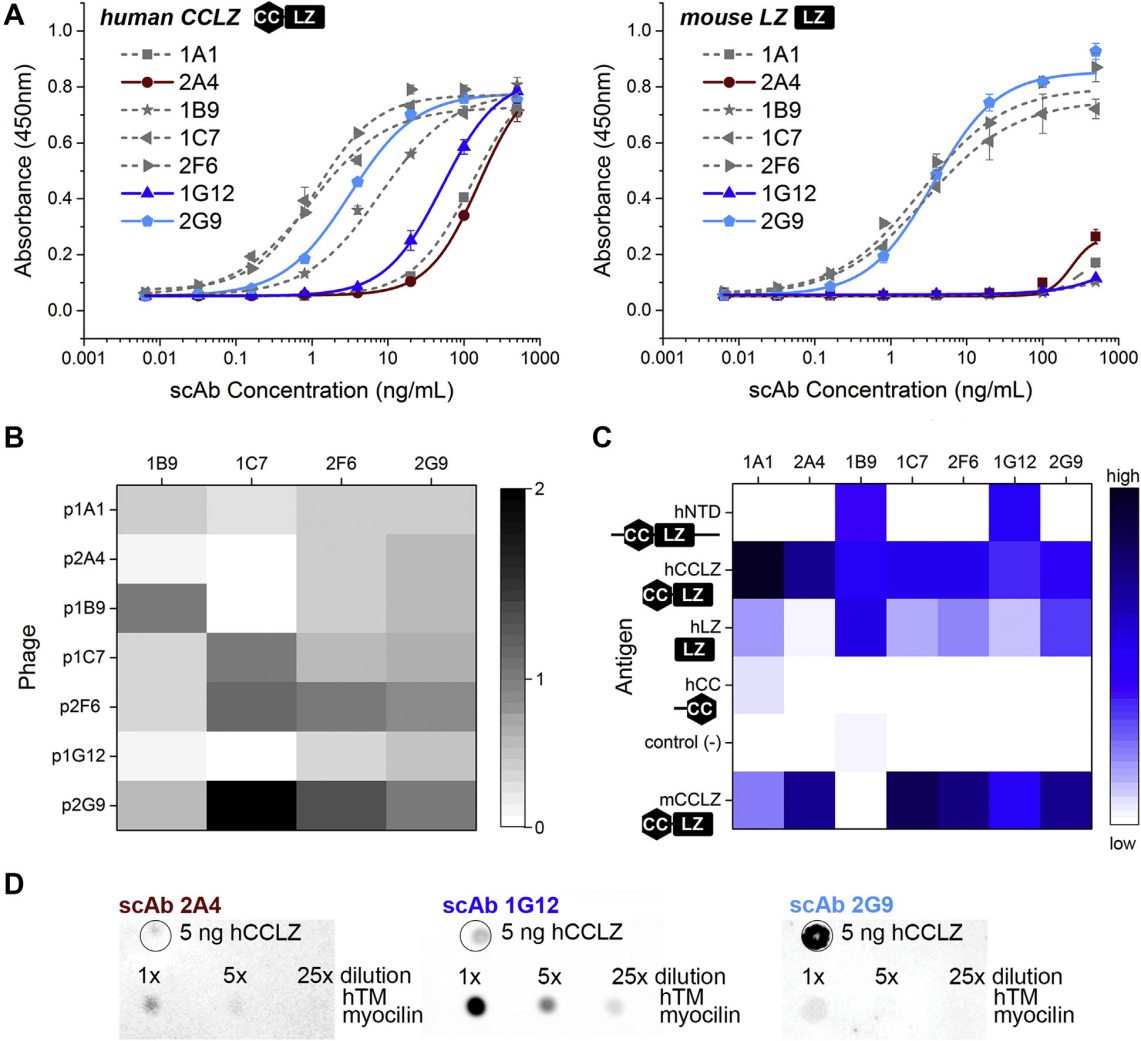
**Evaluation of LZ-targeted scAbs.***A*, binding affinity of candidate scAbs towards hCCLZ and mLZ, with scAbs important for later discussion highlighted with color and solid curves. ELISAs were performed with two technical replicates and were repeated twice. *B*, heat map representation of competition ELISA results wherein values ≥1 indicate competing epitopes. *C*, heat map representation of scAb binding affinity in dot blots against cell lysates containing a range of N-terminal myocilin constructs or a control protein with identical tags. Scale: strongly binding epitopes depicted in darkest blue (high) to nonbinding epitopes white (low) as evaluated in Origin Pro. Data presented are averages of intensities of dot blots with independently prepared lysates against scAbs. *D*, representative of two independent dot blots evaluating scAb binding to heparin-enriched hTM-secreted myocilin.

All 13 candidates expressed well in a soluble scAb format in *Escherichia coli*. Seven of these (1C7, 2F6, 2G9, 1B9, 1G12, 1A1, and 2A4) demonstrated a range of apparent affinities toward purified hCCLZ and mLZ in ELISA. Competition ELISA revealed that clones 1C7, 2F6, and 2G9, which also exhibited the strongest affinities, target the same epitope (Fig. 3, *A* and *B*). Clones 1B9, 1G12, 1A1, and 2A4 appear to target unique epitopes (Fig. 3, *A* and *B*). Six scAbs that exhibited weaker hCCLZ binding in follow-up ELISAs (data not shown) were triaged.

ScAbs 1C7, 2F6, 2G9, 1B9, 1G12, 1A1, and 2A4 were evaluated for their abilities to recognize myocilin constructs in a dot blot, with results summarized as a heat map (Fig. 3*C*). In this technique, the folded antigen is captured on the membrane and then presented to the antibody, analogous to an ELISA but less labor-intensive and higher throughput. We evaluated binding of each antibody to various recombinant myocilin constructs in the context of *E. coli* lysates, as opposed to purified proteins, in order to detect antibodies that lack specificity and react with control lysate. All human myocilin constructs were previously characterized structurally (32); in these experiments, mCCLZ was not subjected to Factor Xa cleavage to remove tags and is therefore intact. All seven scAbs preferentially target LZ (Fig. 1, *A* and *B* and 3*C*), possibly due to inadvertent boosting of the mice with the cleaved construct, mLZ (see above). Surprisingly, the majority of these scAbs only weakly recognize the longer hNTD construct (residues 33–226; Fig. 3*C*). hNTD includes additional linker regions before and after hCCLZ and is conformationally heterogeneous (32), which could affect detection by the LZ-directed scAbs.

To assess whether scAbs recognize full-length myocilin, dot blots were performed using myocilin isolated from the spent media of human TM cells (Fig. 3*D*). For these experiments, myocilin was first enriched by heparin affinity purification. Dot blots using the 2A4 scAb weakly, but reproducibly, detected full-length myocilin. Conversely, dot blots with the 1G12 scAb elicited a strong signal for full-length myocilin, perhaps due to greater availability of its epitope within the protein sample or recognition of a less conformational epitope (see below). The weakest detection of myocilin from human TM cells was observed with scAb 2G9, which was unexpected since 2G9 exhibited one of the strongest binding in ELISA (Fig. 3*A*).

### Conformational specificity of LZ-targeting IgGs

**Figure 4:**
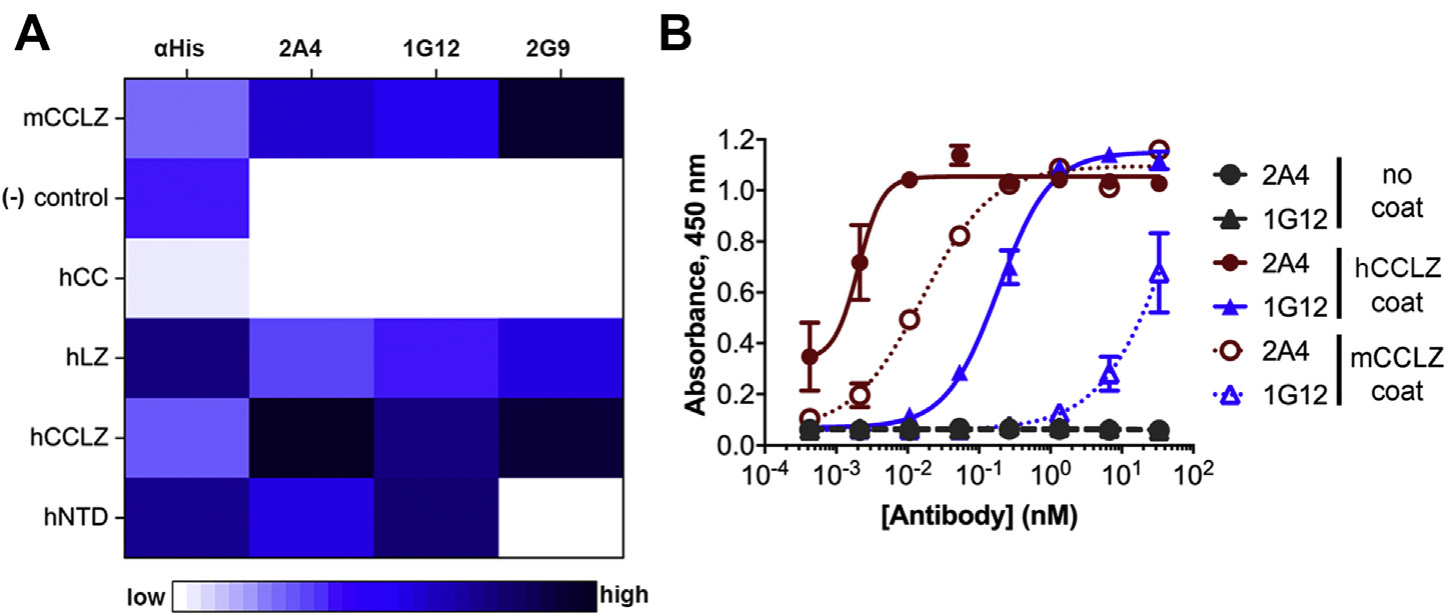
**Evaluation of lead clones as human IgG1 antibodies.***A*, heat map showing reactivity to different myocilin constructs. Scale: strongly binding epitopes depicted in darkest blue (high) to nonbinding epitopes white (low) as evaluated in Origin Pro. See also Figure S2. *B*, ELISA data comparing binding of the two antibodies to human and mouse CCLZ. Experiment repeated three times with duplicates.

Clones 2A4, 2G9, and 1G12 were converted to a human IgG1 format, expressed in CHO cells, and purified using protein A chromatography (Fig. S2*A*). Each IgG was tested for epitope recognition as with scAb format, and each recognizes the same epitope as the respective scAb (Fig. 4*A*, Fig. S2, *B–F*). The IgG format shows increased sensitivity, as expected for the bivalent format. Whereas the scAbs could scarcely detect myocilin in hTM media at a 5× dilution in a western blot, the corresponding IgG showed strong signal with the same media at 100× dilution. It is also notable that 2A4 exhibited considerably more sensitive detection than 1G12 as an IgG, the reverse of that observed with scAbs (Figs. 3*A* and 4*B*).

**Figure 5:**
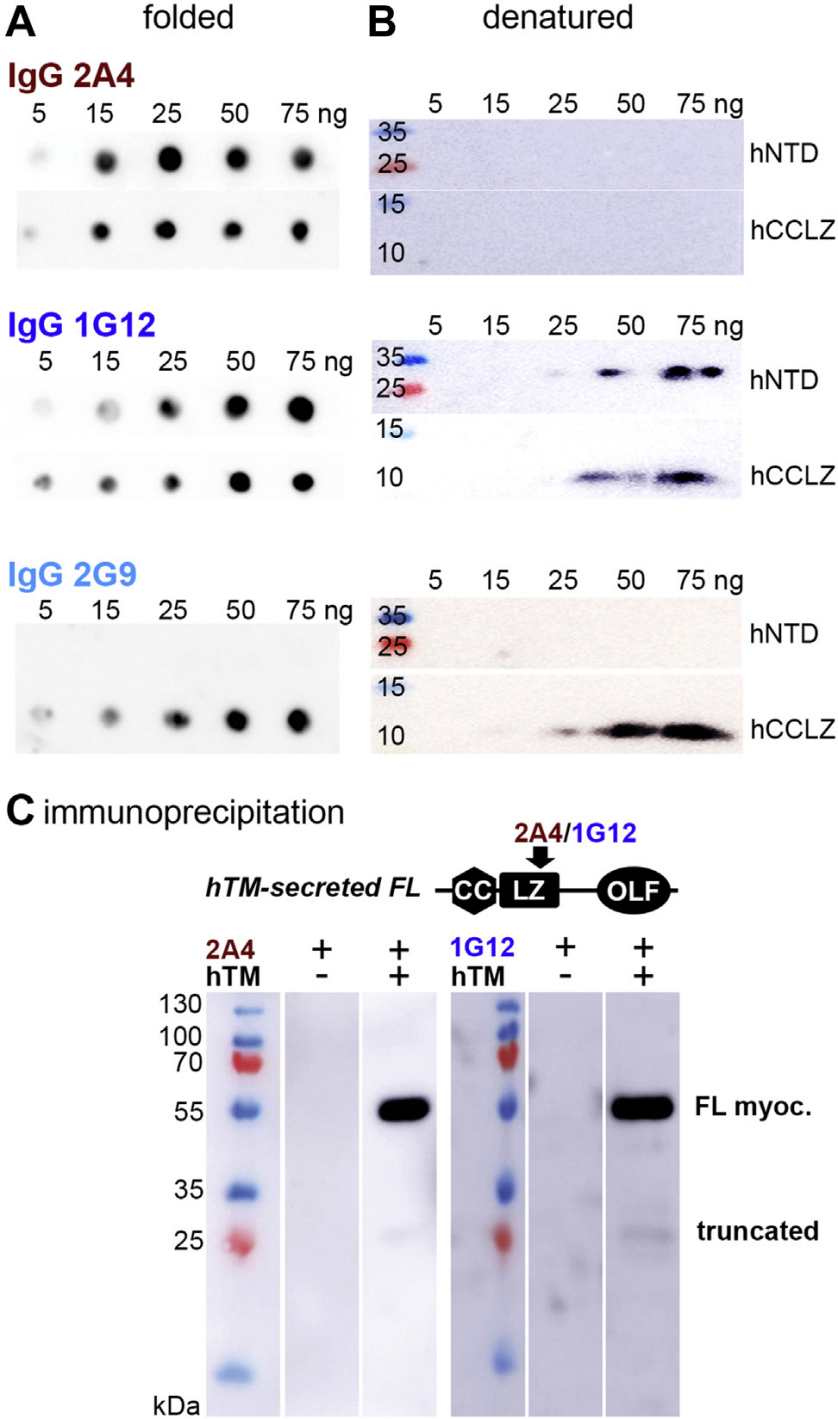
**Conformation selectivity of 2A4 and 1G12.** To assess conformational specificity, myocilin binding of select clones as human IgG1 was assessed by *A*. dot blot and *B*. Western blot against nanogram quantities of purified myocilin. *C*, immunoprecipitation of myocilin from spent hTM media by 2A4 or 1G12 followed by detected in western blot reveals a ∼55 kDa band corresponding to full-length (FL) myocilin, as well as low abundance truncated product of mass consistent with proteolytic products described previously for myocilin (27, 29). See also Figure S3.

Antibody preference for conformational *versus* linear myocilin epitopes was assessed by comparing data from dot blots (Figs. 5*A*, S2, *B–F*, and S3) and dot blots (Figs. 5*B* and S3) in parallel experiments conducted with specific quantities of purified myocilin constructs. Most IgGs displayed robust affinity toward folded hCCLZ and hNTD in dot blots, detecting <15 ng protein. In western blot, where the samples are denatured and disulfide bonds reduced, detection was relatively weak (1G12) or not detectable (2A4). By contrast, 2G9 robustly recognized hCCLZ in both formats, but not longer constructs such as hNTD or full-length myocilin from human TM media. Perhaps the epitope recognized by 2G9 includes the C-terminal Cys185 or a linear epitope that is obstructed in hNTD and full-length myocilin. Thus, at this point 2G9 was removed from further consideration and only 1G12 and 2A4 were characterized further.

### Analysis of myocilin from human TM cell media using 2A4 and 1G12 IgGs

Antibodies 2A4 and 1G12 were tested for their ability to isolate myocilin from spent media of human TM cell culture. All three IgGs immunoprecipitated full-length myocilin, as well as trace amounts of a truncated N-terminal fragment (∼25 kDa) (Figs. 5*C* and S3). Qualitatively, 2A4 and 1G12 immunoprecipitated similar quantities of myocilin.

### Engineering 2A4 for increased stability

**Figure 6:**
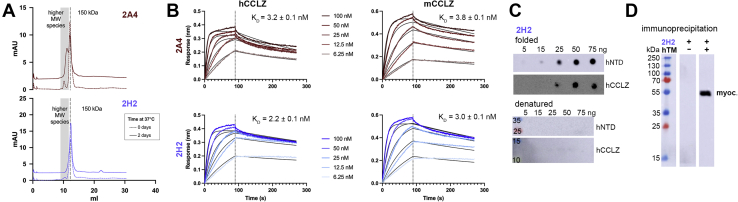
**Comparison of IgG 2A4 and enhanced variant 2H2.***A*, SEC profiles for 2A4 and 2H2 before and after incubation at 37 °C to assess storage stability. Trace is representative of two experimental replicates. *Dotted line marks* 12.3 ml retention, with estimated molecular weight of ∼150 kDa. *Gray shading* indicates the elution volumes for observed higher molecular weight species (9–11.7 ml, ∼215–700 kDa). Day 2 curves are offset 2 mAU on the *y*-axis. *B*, binding to hCCLZ and mCCLZ measured by BLI indicates both antibodies have similar affinities for both ligands. K_D_ values were calculated from k_off_/k_on_ with these rates determined by fitting to a bivalent binding model. Collected data are shown as colored traces, with fits shown as black lines. Errors are SD from globally fitting all concentrations. *C*, conformational selectivity of 2H2 based on dot blot (folded) and western blot (denatured). D. Myocilin immunoprecipitation analyzed by western blot with buffer alone (*left lane*) or spent human TM media (*right lane*). Experiments in *C* and *D* are representative of at least two independent preparations of target antigens. See also Figures S4 and S6

IgGs 2A4 and 1G12 recognize hNTD to similar extents as R&D Systems MAB3446 (Fig. S4*A*), which we judged previously as having the overall best characteristics of commercial antibodies available (31). However, thermal stability measurements for 2A4 and 1G12 revealed that while the Fab domains of 1G12 unfolded at a melting temperature (Tm) indistinguishable from the Fc domain (65–66 °C; Table 2, Fig. S4*B*) and in line with published data for IgGs (33), 2A4 exhibited a surprisingly low Fab domain Tm of 60.6 °C, in a separate transition from Fc (Fig. S4*C*). In addition, we noticed undesirable storage-associated aggregation in multiple purified 2A4 preparations. Namely, size-exclusion chromatography (SEC) elution profiles after a 2 day incubation at 37 °C show that a large fraction of 2A4 (∼40%) elutes as apparent dimers, which is not detected for unheated samples (Fig. 6*A*). Therefore, we next used directed evolution to optimize 2A4 for retention of conformational selectivity but improved biophysical properties.

**Table 3:** CDR H2 library design and sequence comparison of 2A4 and 2H2

IMGT residue #	57	58	59	62	64	66	68	69	70	72
2A4	D	P	E	N	D	E	A	P	K	Q
2H2 (evolved 2A4)	D	P	A	N	S	Y	S	D	K	Q
Library design	Y	Y	Y	Y	Y	Y	Y	Y	Y	K
	A	A	A	A	A	A	A	A	R	Q
	D	D	D	D	D	D	D	D	S	
	S	S	S	S	S	S	S	S	N	
		P	E	N		E		P	V	
		H		T				H	W	
									K

Inspection of the 13 unique sequences recovered from library panning revealed a family of related clones that share similar light- and heavy-chain variable domain sequences (Fig. S1, *B* and *C*). Within this family, the 2A4 CDR H3 loop is unique and extremely short at just three residues, whereas other members have an H3 ranging from 7 to 10 residues. Reasoning that the H3 loop may be largely responsible for 2A4's desirable binding properties but three residues leaves little room for optimization, we instead designed a CDR H2-targeted library. Comparison of CDR H2 sequences within the 2A4 family revealed sequence variation at ten of 16 positions (Fig. S5*A*). We generated a 2A4 library to mimic this diversity while also allowing for the original 2A4 residue at each position. Eight residues were randomized to YADS (34), while greater variation was allowed at position 70 and just K or the original Q at position 72 to yield a theoretical library diversity of 1 × 10^7^ (Table 3). After library generation by PCR amplification with degenerate oligonucleotides and transformation into *E. coli*, the library comprised 2 × 10^8^ clones with 0.04% background. Panning the library for one round on hCCLZ recovered just one novel clone, 2H2, with five residue changes in CDR H2: E59A, D64S, E66Y, A68S, and P69D.

**Table 2:** Biochemical comparison of 2A4 and 2H2

Antibody	Fab melting temp	Fc melting temp	K_D_, hCCLZ (nM)			K_D_, mCCLZ (nM)		
			Kinetic	χ^2^	Equilibrium (95% CI)	Kinetic	χ^2^	Equilibrium (95% CI)
2A4	60.6 ± 0.7 °C	69.3 ± 1.2 °C	3.2 ± 0.1	0.5	4.3 (2.9–5.8)	3.8 ± 0.1	0.6	13.1 (7.4–20.3)
2H2	65.6 ± 0.7 °C	68.7 ± 1.3 °C	2.2 ± 0.1	0.7	6.4 (3.8–9.3)	3.0 ± 0.1	1.0	9.6 (2.6–19.4)

K_D_ measured by BLI with SD shown for kinetic fits and 95% confidence intervals shown in parentheses.

Melting temperatures measured in triplicate in two independent experiments with SD.

After subcloning and purification as an IgG, we compared 2H2 and 2A4 for stability. In the same accelerated aggregation study noted above for 2A4, we incubated 2H2 at 37 °C for 2 days. Heated and control unheated 2H2 both produced a single tight peak with minimal higher-molecular-weight species, as assessed by analytical SEC (Fig. 6*A*). Correspondingly, thermal stability analysis indicates that 2H2 gained ∼5 °C in the Fab Tm relative to 2A4 (from 60.6 to 65.6 °C; Table 2, Fig. S4*C*). Both 2A4 and 2H2 exhibit similar low nanomolar affinities for hCCLZ and mCCLZ as measured by biolayer interferometry (BLI; see Fig. 6*B*, Table 2). The hCCLZ-binding constants (K_D_) were calculated from the on- and off-rates and determined to be 3.2 ± 0.1 nM for 2A4 and 2.2 ± 0.1 nM for 2H2, while binding to mCCLZ yielded values of 3.8 ± 0.1 nM for 2A4 and 3.0 ± 0.1 nM for 2H2. The K_D_ was also measured by steady-state analysis (Table 2, Fig. S7) and found to be similar. Due to the technical limitations of BLI measurements for high-affinity interactions (35) and the complex valency of this specific interaction, these values are considered observed K_D_ values to guide future experiments. 2H2 exhibited no evidence of polyspecific binding to a panel of ELISA antigens with diverse biochemical features (Fig. S5*B*). Overall, 2H2 has markedly improved stability compared with 2A4, while retaining low-nM cross-reactive binding to human and mouse CCLZ.

### Conformational selectivity of 2H2 and application to fluorescence imaging of fixed human TM cells

To evaluate whether affinity-matured antibody 2H2 retains the conformational selectivity of 2A4, western blots and dot blots were performed with purified hNTD and hCCLZ, as described above. Similar to 2A4 (Fig. 5), 2H2 was able to detect folded myocilin constructs in dot blots at low ng amounts while remaining unable to detect 75 ng denatured protein in a western blot (Fig. 6*C*). Immunoprecipitation with spent media of human TM cells revealed that 2H2 is also able to recover full-length myocilin (Fig. 6*D*).

**Figure 7:**
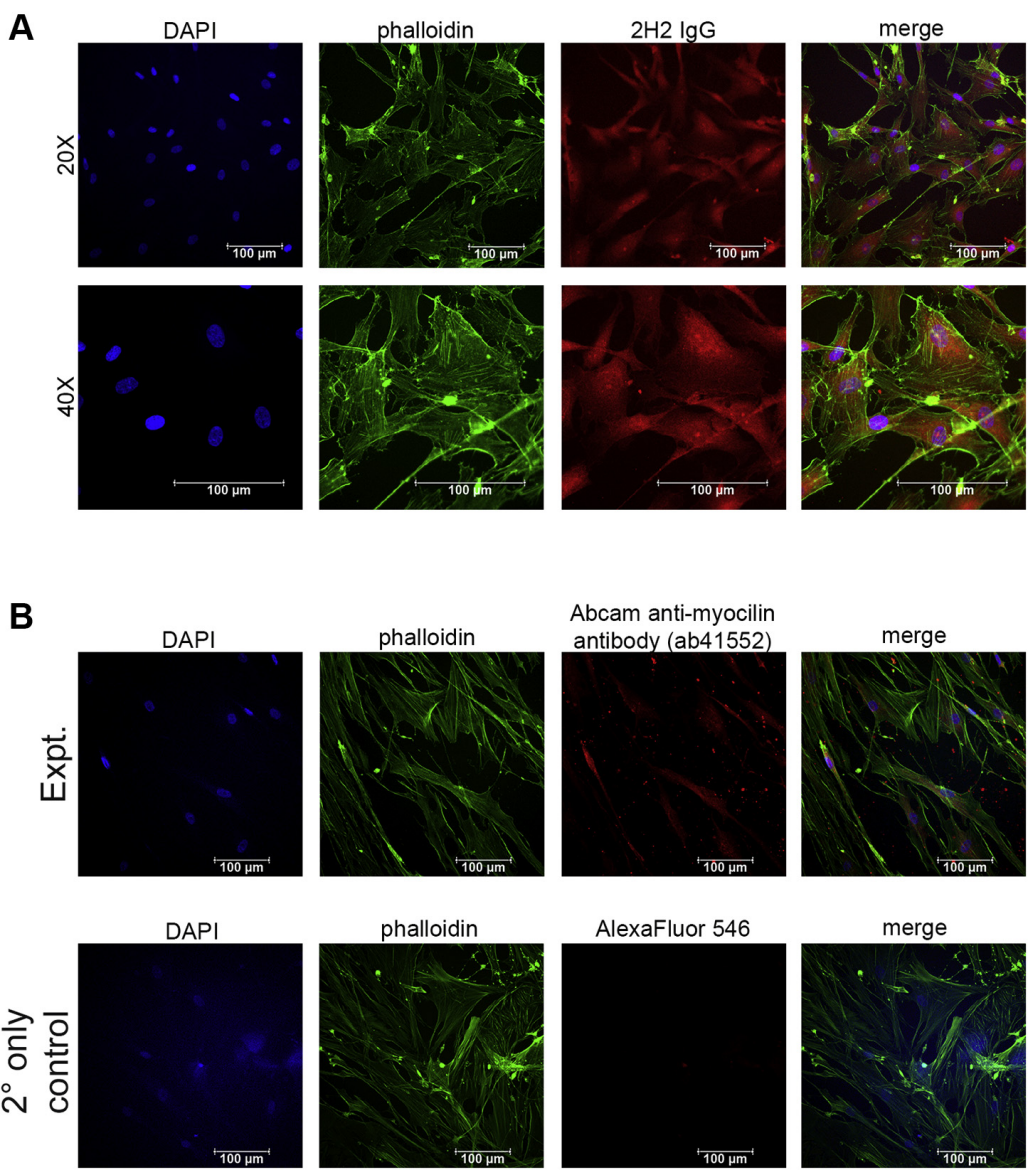
**Immunostaining of fixed human TM cells.***A*, individual panels for nuclear staining (DAPI), actin filament staining (phalloidin), Alexafluor647-conjugated 2H2 (1:200), and merged image at two magnifications. *B*, individual panels for Abcam anti-myocilin antibody (ab41552, 1:100) experiment and secondary antibody-only control: nuclear staining (DAPI), actin filament staining (phalloidin), anti-myocilin Abcam antibody, and merged images. Representative images from two cover slips.

To determine the potential to use 2H2 to address research questions in clinical samples, we assessed the ability of 2H2 to stain myocilin in fixed human TM cells (Fig. 7). We directly conjugated 2H2 with the fluorophore AlexaFluor 647 to prevent the nonspecific binding that can occur from an antihuman secondary antibody staining of human cells. Fluorescence imaging with 2H2 revealed myocilin as expected in the secretory pathway (Fig. 7*A*). By contrast, 2H2 shows no binding to a human cell line that does not express myocilin when used at concentrations as high as 250 nM (Fig. S5*C*). Staining of myocilin by 2H2 at a higher dilution factor produced a similar pattern to that of commercial myocilin antibody ab41552 (Table 4, Fig. 7*B*) (36, 37), which we previously showed recognizes myocilin species containing the far-N terminal residues just before the structural CC domain (Fig. 1*A*) (31). The cellular location of myocilin by 2H2 is also similar to that of OLF-directed (Fig. 1*A*) commercial myocilin antibodies from Santa Cruz (38, 39). We note that direct comparison across samples is a challenge because myocilin levels in primary cells from different individuals may not be the same, antibody dilutions for immunostaining are not always reported, and published images are often after dexamethasone treatment, which stimulates myocilin expression in human TM cells (15). Nevertheless, taken together, these data support the feasibility of using 2H2 to support histological research on patient-derived samples.

**Table 4:** Commercial antibodies used in this study

Antibody	Identification	Epitope & Clonality
Commercial anti-LZ	R&D Systems Cat# MAB3446 RRID: AB_2148649	Human myocilin LZ within residues 112–185 (monoclonal)
Commercial myocilin antibody for immunostaining	R&D Systems Cat# ab41552 RRID: AB_776605	Human myocilin residues 25–45
Mouse anti-His *4A12E4*	Fisher Scientific Cat# 37-2900 RRID: AB_2533309	Hexahistidine Tag (monoclonal)
M13 *RL-ph1*∗HRP	Santa Cruz Cat# sc-53004 RRID: AB_673750	M13 phage coat protein (monoclonal∗HRP)
Mouse anti-myc *9E10*	Santa Cruz Cat# sc-40 RRID:AB_627268	human C-terminal Myc residues 408–439 (monoclonal)
Goat anti-mouse ∗HRP	Thermo Fisher Cat# 62-6520 RRID: AB_2533947	mouse IgG (polyclonal∗HRP)
Goat anti-human κ ∗HRP	Southern Biotek Cat# 2060-05 RRID: AB_619883	human kappa light chain (polyclonal∗HRP)

Antibody conjugation is indicated by an asterisk, and clone designations are italicized.

## Discussion

Inspired by the advances in other protein misfolding diseases that have been catalyzed by the availability of conformation-dependent and recombinant antibody reagents (6, 7, 8, 9, 10, 11, 40, 41, 42), we aim to ultimately develop a toolbox of new high-quality antibodies for myocilin, a relatively new addition to the list of proteins associated with misfolding diseases. As a first effort, we identified antibodies recognizing the N-terminal region, which is composed of previously characterized helical domains (32). We immunized mice with purified mouse and human N-terminal myocilin protein constructs, followed by an *in vitro* antibody selection strategy. Reasoning that antibodies binding conformational epitopes on properly folded myocilin may be rare, we designed our initial immune library generation and selection process to recover a diverse set of antibodies. To support this goal, we generated a large immune library (1.5 × 10^7^ clones) and characterized clones after the second and third rounds of myocillin selection to identify 13 candidates. Further screening identified antibodies 2A4 and 1G12 that bind different conformational epitopes in the LZ domain. We then engineered 2A4 for improved biophysical properties, resulting in clone 2H2. Antibodies 2A4 and 2H2 recognize LZ only in its folded form, suggesting they target a discontinuous epitope, while antibody 1G12 weakly detects denatured proteins in western blot, indicating that it recognizes a mostly conformational epitope with a linear component. These are all able to immunoprecipitate myocilin from the spent media of human TM cells and 2H2 detects myocilin in fixed human TM cells.

A constraint during selection was identification of antibodies that bind the human and mouse CCLZ isoforms with similar affinities. This is an important consideration since it allows for the same antibody to be used in experimental mouse models and human tissues, thereby facilitating comparison between these experiments and supporting extrapolation of animal model data to humans. In the absence of cross-reactive antibodies, often two parallel antibody discovery campaigns are pursued, resulting in identification of two antibodies that each bind a similar epitope on the homologous antigen. Not only does this double the effort required for antibody discovery, but depending on the antibody fine epitope specificity, the impact of the antibody on disease in the different model systems may not be identical. This has been a challenge in developing antibodies to bind mouse CD3 to evaluate T cell retargeting antibodies and to evaluate antibody therapeutics to treat pediatric medulloblastoma in mice (43, 44, 45). In support of this future effort for myocilin, 2H2 has similar (within ∼50%) K_D_ values for each homolog and can easily be expressed with mouse or human constant domains to faithfully engage host Fc receptors (Table 2).

The finding that the antibodies reported here bind LZ from human and mouse homologs suggests they recognize a region with conserved structural or sequence features. Indeed, not only do human and mouse LZ share high levels of sequence similarity but given the similarity of myocilin across commonly used glaucoma animal model systems including monkey, pig, and cat, (∼50% identical, 88% similar across nine species, Fig. 1*C*), these epitopes may be shared on these homologs as well. Notably, human and mouse LZ bracket the range of calculated pI values of 5–9 across inspected LZ sequences, indicating that the recognition epitope is likely outside regions harboring changes in charged residues. Additional characterization and, as needed, antibody maturation steps are also possible to expand antibody applicability to additional species, as reported for pollen antigens (46). Cross-reactivity of myocilin antibodies broadens our ability to investigate the roles of myocilin aggregation and secretion across species.

Antibodies 1G12 and 2A4 are interesting because neither was strongly selected during library panning; each CDRH3 sequence appeared just once out of 80 clones sequenced (Fig. S1*B*). By contrast, 2G9 was the second most strongly selected clone, represented in 16% of sequences, but binds a less useful epitope in mouse and human LZ that is obscured in longer constructs (Fig. 5). Antibody selection strategies, including bacterial, phage, and yeast display, are biased to select for clones that fold well and impose minimal growth defects when expressed in the display system while tightly binding accessible epitopes on the ligand. Antibody 2G9 meets these selection criteria by binding an accessible but undesirable epitope. This seems to be a common feature of antibody selection campaigns against aggregation-prone antibodies (7), perhaps because these epitopes are readily available in the ensemble of protein conformations available during selection. Based on our analysis of 2A4, its poor thermal stability and weak binding in the scAb format also likely contributed to its weak enrichment during panning and may have conferred a growth disadvantage relative to other antibodies during library propagation. Since the constraints imposed during antibody phage selection are different from those experienced during expression of an intact IgG, our less-stringent selection process that preserved clonal diversity was key to identification of 2A4. Future strategies to select for antibodies binding conformational epitopes in aggregation-prone proteins may benefit from selection steps that remove antibodies binding less-desirable conformers, for instance, by homogeneous, properly folded protein or linearized domains.

A second intriguing feature of the 2A4 antibody is that it binds weakly as a monovalent scAb but very tightly as a bivalent IgG (∼1000-fold better on ELISA; Figs. 3 and 4). Affinity is expected to scale with valency, since the on-rate is statistically related to the binding site surface area while off-rate can be reduced through rebinding and avidity effects (47, 48, 49). For instance, the transition from monovalency to bivalency would be expected to increase the on-rate by a factor of 2, since the ligand is twice as likely to collide with an antibody-binding site. However, these effects do not explain the dramatic increase in bivalent binding observed, suggesting other phenomena are involved. An allosteric or cooperative binding effect may be dependent on the bivalent format, such as simultaneous engagement of two LZ epitopes on the tetrameric myocilin protein (Fig. 1*B*) or induction of a conformational change during binding of the first Fab arm, which facilitates binding of the second Fab arm. This phenomenon was also observed for an engineered antibody binding a conformational epitope at the extreme N-terminus of human Aβ fibrils (50).

To remedy the lackluster storage and stability features of 2A4 while retaining conformational selectivity, we used directed evolution of CDRH2 to identify the improved antibody 2H2. Variant 2H2 has five residue changes that overall reduce the negative charge of this loop (E59A, D64S, E66Y) and increase its flexibility (P69D) while retaining similar affinities for human and mouse CCLZ (Tables 2 and 3). Since these changes dramatically reduced formation of higher molecular weight species under mildly stressful conditions (Fig. 6*A*), we speculate that the decreased surface charge may reduce short-range electrostatic forces that can mediate undesirable protein–protein interactions at high concentrations (51). A general conclusion is that selection of antibodies binding conformational epitopes in aggregation-prone proteins may benefit from a two-step selection process, as was used here: (i) identification of an antibody binding an epitope of interest from a diverse group of antibodies, followed by (ii) optimization of antibody affinity, stability, and other biochemical features.

Our LZ-directed antibodies, and those we expect to isolate recognizing epitopes in the CC and OLF domains in future work (Fig. 1, *A* and *B*), will enable us to expand our understanding of the breadth of conformational states adopted by myocilin. Numerous conformational and oligomeric states, largely of unknown functional or disease significance, are documented for myocilin. For example, proteolytic cleavage of myocilin (27, 29, 52), which separates OLF from the N-terminal coiled domains, may play a functional role, as it does for olfactomedin family-members gliomedin (53, 54) and latrophilin (55). In addition, myocilin has been identified in a variety of disulfide-mediated oligomerization states, ranging from dimer (29, 56) to tetramer and higher (28, 32, 57). Glycans in a presumed disulfide-shuffled myocilin oligomers are resistant to removal by PNGase F, suggesting a significant structural change and possible functional role in redox signaling (29). Regarding association with glaucoma, dominant-inherited single-point mutations in the OLF domain of myocilin lead to its facile aggregation into amyloid-like fibrils (58, 59), a significant source of conformational heterogeneity.

The importance of understanding conformational heterogeneity is underscored by the primary pathogenic mechanism by which full-length mutant myocilin misfolds intracellularly, leading to a toxic gain of function—TM cell death—that ultimately hastens the timeline for glaucoma-associated IOP elevation and vision loss (60). Future availability of conformational antibodies directed toward different parts of the OLF domain will be very useful in evaluating accessibility of a given myocilin epitope in different biological and pathogenic contexts including but not limited to myocilin mutations (18), steroid treatment (15), mechanical stress (21), and oxidative stress (61, 62). In the long term, a better understanding of myocilin in its myriad alternative states will improve our molecular comprehension of a number of human tissues and associated diseased states and offer new options for diagnostics and treatments.

## Experimental procedures

### Expression and purification of myocilin constructs

N-terminal myocilin fragments (see Table 1) were expressed in *E. coli* BL21-DE3-pLysS using pET-30 XaLIC (32) or in pMAL (hNTD, comprising residues 33–226, only) (31) vectors. Proteins were purified as described previously (32) by nickel affinity chromatography followed by size exclusion on AKTA Pure or Purifier (GE Healthcare). For immunization and panning, expression tags were cleaved using Factor Xa (New England Biolabs) and purified proteins isolated by heparin affinity and SEC (32). Protein concentrations were determined by absorbance at 280 nm using ExPaSy-predicted extinction coefficients, as described previously (32).

Our intent was to immunize mice with hCCLZ (comprising residues 69–185) and boost with the orthologous mouse construct mCCLZ (residues 55–171). However, after immunization, we learned that during purification our mCCLZ construct is cleaved by Factor Xa at a fortuitous site (TLGR_92_, Fig. 1*C*) (32). Thus, the actual boosting immunogen used was mLZ (residues 93–171). Similar secondary sites have been reported in literature exploring Factor Xa specificity (63, 64).

For dot blots, cell lysates were prepared by sonicating 0.2 g cell paste in 1 ml buffer (50 mM HEPES pH 7.5, 200 mM NaCl, 10% glycerol) until clarified, then the insoluble material was removed by centrifugation at 17,000*g* (31). Cell lysates used for the negative control were derived from BL21-DE3-pLysS cells expressing Grp94, which possesses the affinity tags present in the N-terminal myocilin constructs used here. Full-length endogenous myocilin used in dot blots was enriched from human TM spent medium by heparin affinity, also as described previously (31).

### Mouse immunization

All protocols were approved by the University of Texas at Austin IACUC (AUP-2018-00092), and all mice were handled in accordance with IACUC guidelines. BALB/c mice (6 weeks old, n = 3) were immunized subcutaneously with 5 μg of hCCLZ in Freund's adjuvant. Four weeks later, mice were boosted subcutaneously with 5 μg of mLZ (see above) using incomplete Freund's adjuvant. Two weeks later, mice were sacrificed, their spleens harvested and stored in 1 ml RNAlater solution at –80 °C. Collected sera were used in ELISA to determine the serum antibody titers against hCCLZ and mLZ.

### Phage display antibody library construction

Total RNA was extracted from frozen spleens with TRIzol (Invitrogen) and the PureLink RNA kit (Invitrogen) according to the manufacturers' instructions, with the RNA concentration determined using a NanoDrop2000 (Thermo Scientific). First strand cDNA synthesis was performed using 500 ng RNA and the SuperScript IV Transcriptase (Invitrogen) kit according to the manufacturer's instructions and stored at –20 °C until further use.

The variable heavy (V_H_) and variable light (V_L_) chain repertoires for each mouse were amplified from cDNA using the primer sets and PCR conditions described previously (65). The V_H_ and V_L_ fragments were then used as a template (30 ng of each) for an overlap PCR (65) to generate single-chain variable fragments (scFv) in the V_L_-linker-V_H_ orientation. The overlapped PCR fragments (about 10 μg per mouse) were gel purified using Zymoclean Gel DNA Recovery kit; this product and freshly purified pMopac24 plasmid (50 μg) were digested using *Sfi*I restriction enzyme (NEB) overnight at 50 °C. The digests were gel purified and cleaned using Zymoclean Gel DNA Recovery kit. Ligation of the vector and insert was performed using T4 DNA ligase (NEB) (400–500 μl ligations per mouse) at 16 °C overnight. The next morning (after 16–18 h), the ligation was heat inactivated for 20 min at 65 °C and all ligations were pooled and concentrated 10-fold using n-butanol. The concentrated ligation was then desalted using nitrocellulose membranes (Thermo Fisher Scientific) for 2 h before electroporating into freshly prepared XL1Blue electrocompetent cells (30–40 electroporations). Transformed cells were recovered in warm SOC medium for 1 h before plating on medium 2xYT with 1% glucose and 100 μg/ml ampicillin (2xYT+GA) agar plates. An aliquot was 10-fold serially diluted and plated to determine library size. Plates were left overnight at room temperature and scraped the next morning into 2xYT+GA. The scraped bacteria were pooled to form the master library and frozen in 1 ml aliquots at an OD_600nm_ = 5 at –80 °C.

### Phage production, purification, and panning

One library aliquot was thawed and used to inoculate a 30 ml 2xYT+GA to a starting OD_600_ of 0.08–0.1. The flask labeled “input” was then grown at 37 °C shaking at 225 RPM for 2–3 h until the OD_600_ was between 0.4 and 0.6. At this time, media was adjusted to 1 mM IPTG to induce scFv expression and M13KO7 helper phage added at a multiplicity of 20. The flask was allowed to shake an additional 30 min at 37 °C before reducing the temperature to 25 °C. Three hours after adding the helper phage, the culture was supplemented with 25 μg/ml kanamycin and allowed to shake overnight at 25 °C. Phage were purified from the culture supernatant using one fifth volume of precipitation solution (2.5 M NaCl and 20% PEG-8000). Phage were titered by adding serially diluted phage to mid-log phase XL1Blue cells before plating on 2xYT+GA plates to determine the phage forming units (pfu).

Eight ELISA plate wells (Corning) were coated with 50 μLs of 2 μg/ml anti-c-myc (9E10; BioXCell) monoclonal antibody, hCCLZ protein, or mLZ protein each in PBS overnight at 4 °C. The library was first panned against anti-c-myc antibody to enrich full-length scFvs for subsequent rounds of panning. Panning was then performed on immobilized hCCLZ and mLZ antigens using the schematic shown in Figure 2 to isolate cross-reactive scFvs. ScFv sequence diversity was monitored throughout all the steps by colony PCR and *Bst*NI fingerprinting as well as Sanger Sequencing.

Monoclonal phage ELISAs were performed on sequence-unique clones with diverse CDRs. Cultures of single clones (2 ml) were grown to an OD_600_ = 0.5 in 2xYT+GA, used to produce recombinant phage as described above, and resuspended in 200 μl PBS. ELISA plates were coated with 2 μg/ml antigen (hCCLZ or mLZ) diluted in PBS overnight at 4 °C, plates blocked and phage diluted 1:3 with PBS-milk and titrated as the primary stain. Bound phage were detected using a 1:2000 dilution of mouse anti-M13-HRP antibody (Table 4) in 1xPBS, 0.1% Tween-20, 5% milk.

### 2A4 library design and selection using phage display

A library was generated by amplification of 2A4 with degenerate primers and an overlapping PCR scheme to alter selected CDRH2 positions to wild-type or the residues shown in Table 3. The light chain, linker, and heavy chain were amplified with 5′ scback (5′-TTACTCGCGGCCCAGCCGGCCATGGCGGACTACAAAG-3′) and 3′ CDRH2 reverse (5′-AATCCATCCAATCCACTCCAGGCCCTGTTCAGGCCTCTGCTTCACCCA-3′) while degenerate codons were introduced with 5′ CDRH2 forward (5′-CTGGAGTGGATTGGATGGATTKMTBMTKMKDMTGGTKMTACTKMKTATKMTBMTWRSTTCMAAGGCAAGGCCACTATG-3′) and 3′ scfor (5′-GGAATTCGGCCCCCGAG-3′). The fragments were purified, digested with *DpnI* (NEB) to degrade template DNA, and overlapped using a two-stage PCR with primers scfor and scback. In the first stage, the two fragments were annealed and extended at an equimolar ratio for 25 cycles with an annealing temperature of 63 °C and without added primers. In the second stage, primers were added and the scFv was amplified for 32 cycles with an annealing temperature of 72 °C. The 750 bp product and fresh purified pMopac24 plasmid were *Sfi*I digested, ligated, concentrated, desalted, and electroporated as described above. Phage production and purification proceeded as described above. The library was panned against anti-c-myc antibody and then on immobilized hCCLZ antigen. Sanger sequencing of ten random colonies after each panning step established that after this first hCCLZ selection, 60% of sequences had converged back to wild-type, and so panning was stopped to prevent loss of diversity.

### Antibody expression and purification

#### scAb production

To produce soluble scAbs, scFvs were subcloned from pMoPac24 *via* directional *Sfi*I (NEB) digestion to the compatible pMopac54 vector, which produces soluble scFv protein followed by a human constant kappa sequence and a His_12_ tag. After sequence verification, plasmids were transformed into BL21/DE3 for expression. Starter cultures were used to inoculate 250 ml TB with 1% glucose and 100 ug/ml ampicillin and were allowed to shake overnight at 37 °C and 225 rpm. Cultures are harvested by centrifugation at 5000 rpm for 10 minutes, pellets resuspended in 250 ml fresh TB supplemented with 100 ug/ml ampicillin. After 1 h shaking at 25 °C, expression was induced with 1 mM IPTG for an additional 5 h. Osmotic shock to harvest the periplasmic proteins was performed as previously described (66). ScAbs were then purified using IMAC resin followed by SEC with a Superdex 75 column on an Åkta FPLC (GE Healthcare). Protein concentrations were measured using Nanodrop 200 and Expasy-derived extinction coefficients for each construct. Purity was assessed by SDS-PAGE on a 4–20% gel (Bio-Rad) with Coomassie staining.

#### Recombinant IgG production

Select scFvs were converted into chimeric human IgG1 antibodies by cloning the V_H_ and V_L_ domains with primers into Igκ-Abvec and IgG-Abvec vectors as described previously (67). Plasmids were transfected into ExpiCHO cells using a 4:1 ratio of light to heavy chain vector and ExpiFectamine (Thermo Fisher Scientific) according to the manufacturer's standard protocol. Transient expression proceeded using ExpiCHO Expression Media (Thermo Fisher Scientific) and supplemented with Expifectamine CHO Enhancer and Expifectamine CHO Feed after 1 day. Cells were maintained at 37 °C for 1 week with shaking and then cell culture supernatant was collected and applied to a HiTrap protein A column and eluted using a 100 mM sodium citrate and 50 mM sodium chloride pH 3.0 elution buffer. Protein concentration and purity were assessed as above.

### ELISAs

ELISAs with purified scAb or IgGs were performed by coating high binding plates (Corning) with 1 μg/ml mLZ, hCCLZ, or hCC overnight in PBS at 4 °C. The following day, plates were blocked with PBS supplemented with 5% milk and 0.1% Tween-20 (blocking buffer) for 1 h at room temperature. Antibodies (scAb or IgG) were serially diluted (1:5) in blocking buffer to the blocked plates. Secondary antibodies were added at 1:1000 dilution of either goat anti-human κ∗HRP for scAbs or goat anti-human IgG Fc HRP for full-length antibodies. Signal was developed using TMB solution (Thermo Scientific Pierce), quenched with 1 N HCl and measured at 450 nm.

For competition ELISAs, scAbs were serially diluted from 50 μg/ml in the presence of a constant concentration of scFv-displaying phage (1:1000–1:3000 dilution of precipitated phage; this concentration was empirically determined by prior phage ELISA) in blocking buffer. Bound phage were detected using 1:2000 dilution of mouse anti M13 HRP antibody (Table 4) in blocking buffer; signal was developed and recorded as above. The 50% inhibitory concentration (IC_50_) for each scAb was calculated using GraphPad prism and compared with controls (antibody competition with itself). After importing the normalized data, heat maps were generated in Origin Pro 2016 using the heatmap feature.

### Storage stability assay

Recombinant IgG was purified by SEC prior to aggregation studies. Using a Superdex 200 Increase 10/300 Gl column (GE Healthcare), the peak eluting at ∼12.3 ml (estimated molecular weight 165 kDa) corresponding to heterodimeric IgG was collected. Aliquots at a concentration of 0.8 mg/ml and a total volume of 125 μL of each antibody were prepared and incubated at 37 °C for 2 days in a heated lid incubator or kept as 4 °C as controls. These were assessed for extent of monomer present by analytical SEC on the same S200 column.

### Bio-layer interferometry

K_D_ values were obtained using the OctetRed (ForteBio) instrument. Ni-NTA sensors (Sartorius) were loaded with CCLZ constructs in HBS-EBT buffer (10 mM HEPES, 150 mM NaCl, 3 mM EDTA, 1 mg/ml BSA, and 0.05% Tween-20) to a final 0.5 nm shift. Sensors were blocked using HBS-EBT buffer with 0.2% casein and seven sensors were allowed to bind 2A4 or 2H2 hIgG in HBS-EBT for 90 s at concentrations ranging from 400 nM to 6.25 nM in 2-fold serial dilutions. As a control, an eighth sensor was dipped in HBS-EBT with no antibody for 90 s. Sensors were then dipped into HBS-EBT for 180 s to monitor antibody dissociation. Reference subtracted curves for concentrations 100–6.25 nM were used to fit a bivalent kinetic binding model using the ForteBio analysis software. Reference subtracted curves that reached equilibrium were also averaged between 80 and 88 s to obtain the equilibrium response for steady-state analysis. These were then globally fit to the “one site specific binding” equation on GraphPad Prism to obtain dissociation constants (K_D_) and their associated asymmetric confidence intervals.

### Dot and western blots

Dot and western blots were conducted using protocols previously described (31). Protein samples were immobilized on methanol-activated polyvinylidene difluoride membranes (Millipore) by electrophoresis for western blots and by direct deposition of 2 μl sample per dot for dot blots. Membranes were blocked overnight in 2% milk in PBS supplemented with 0.5% TWEEN-20, then probed with primary antibodies. Purified scAbs were evaluated at 1 μg/ml; IgGs were tested at 0.5 μg/ml to account for bivalency. Protein expression in cell lysates was confirmed using 1:1000 mouse anti-His antibody (Table 4). Commercial anti-LZ antibody (0.5 μg/ml, Table 4) was used as a positive control. Custom scAbs and IgGs were detected with 1:2000 (whole cell lysate dot blots) or 1:5000 (all other experiments) dilution of HRP-conjugated goat anti-human κ light chain secondary antibody (Southern Biotek, Table 4). Binding of control antibodies was detected using 1:2000 (whole cell lysate dot blots) or 1:5000 (all other experiments) dilution of HRP-conjugated goat anti-mouse secondary.

Following application of primary and secondary antibodies, membranes were treated with HyGlo Quick Spray (Denville Scientific) and imaged using either an Amersham Imager A600 (GE Healthcare) or a ChemiDoc MP Imaging System (Bio-Rad). Western and dot blot results represent at least two biological replicates per antibody tested. Heat maps were generated by measuring the intensity of a 25-fold diluted cell lysate dots using the Gel Analyzer function within ImageJ Gel (68). Heat maps were generated in Origin Pro 2016, and represent the average of four measurements across two biological replicates.

### Differential scanning fluorimetry

Melting temperatures were determined by differential scanning fluorimetry using Sypro Orange (Invitrogen) and 100 μM IgG in 50 mM sodium phosphate pH 7.2, 150 mM NaCl (PBS). Samples were prepared on ice and dispensed into 96-well optical plates (Applied Biosystems) in triplicate, 20 μl per well. Thermal melts were performed from 25 to 95 °C with a 1 °C per min ramp rate using a Thermo Fisher Scientific Viia7 RT-PCR instrument with fixed excitation wavelength (480 nm) and ROX emission filter (610 nm). Values represent an average of triplicate samples from two separate experiments with sample standard deviation.

### Immunoprecipitation from human TM spent media

Spent media from primary human TM cells were a kind gift from E. Snider and C. R. Ethier (69). For dot blots in scAb format (Fig. 3*D*), myocilin from human TM cells was first enriched by elution from a heparin column. Briefly, a 5 ml HiTrap Heparin column was equilibrated with 10 mM Na_2_HPO_4_/10 mM KH_2_PO_4_ buffer pH 6.8, 200 mM NaCl. After the spent media was applied and baseline was reached, an elution gradient from 0.2 to 1 M NaCl was applied. Myocilin eluted in fractions containing ∼400 mM NaCl, as confirmed by western blot (not shown). For immunoprecipitation experiments antibodies in IgG format (0.5 ng/ml) were incubated with 10 ml spent human TM medium (experiment) without prior enrichment, or 10 ml buffer control (50 mM HEPES pH 7.5, 200 mM NaCl, 10% glycerol) overnight at 4 °C on a rocker. Subsequently, Pierce Protein A/G Plus agarose suspension (50 μl, Thermo Fisher Scientific) was added, and samples were rocked an additional 2 h at room temperature. Resin was pelleted by centrifugation (10 min at 1000*g*), washed at least five times with 500uL buffer. Bound proteins were eluted by adding 50 μl 2× Laemmli buffer, incubated for 10 min at room temperature, followed by pelleting and removing the supernatant. Samples were supplemented to 3% v/v β-mercaptoethanol and heated 10 min at 95 °C prior to SDS-PAGE and western blot analysis (1:1000 R&D Systems anti-LZ primary antibody) and 1:5000 goat anti-mouse κ∗HRP secondary (Table 4). At least two biological replicates were performed per antibody tested.

### Fluorescence imaging

2H2 was conjugated to Alexa Fluor-647 using the standard protocol of the Alexa Fluor 647 Antibody Labeling Kit (Invitrogen A20186), achieving a final degree of substitution of 2.8 mol Alexa Fluor-647/mol 2H2. Human TM cells used in fluorescence imaging were validated and generously donated by W.D. Stamer (Duke) (70, 71). For imaging with 2H2 IgG1, cells at passage 6 were seeded on coverslips treated with poly-L-lysine (2 μg/cm^2^). Cells were cultured to 80–95% confluency and fixed with 10% formalin. For immunostaining, cells were incubated in permeabilization/blocking buffer (“PB”; 10% goat serum, 0.1% Triton in PBS) for 1 h at room temperature. PB buffer was removed and cells were incubated with engineered antibody 2H2 conjugated to Alexa Fluor λ647 (1:200 dilution or ∼125 nM, in PB buffer with DAPI and phalloidin λ488) overnight at 4 °C in total darkness. Cells were washed 3× with PB buffer at room temperature (20 min for each wash). Coverslips were mounted on Superfrost Plus microslides (“ProLong Gold antifade reagent”; Invitrogen #P36934) and mounting medium was allowed to dry for 24 h. For imaging with Abcam anti-myocilin (ab41552, 1:100), a second primary human TM cell line at passage 5 from the Stamer lab was used. These cells were seeded on coverslips treated with 0.1% gelatin and grown to ∼90% confluency. The secondary antibody was goat anti-rabbit IgG Alexa Fluor λ546 (Invitrogen), otherwise the same protocol for fixing, permeabilization, and overnight primary antibody incubation was followed as for 2H2. The next day the primary antibody was removed, samples washed 3× with PB for 10 min, and the secondary antibody solution (1:200) is prepared in PB with DAPI and Phalloidin λ488 was applied to the cover slips. After a 3 h incubation at 4 °C in total darkness, the solution was removed, washed with 3× with PB for 10 min, and then the cover slips were mounted onto slides as above. For the secondary only control, the same protocol was followed except PB was added without primary antibody. Cells were imaged using a Leica DM6 microscope and the images processed using “Leica Application Suite X” software.

## Data availability

Raw data will be made available upon reasonable request.

## Supporting information

This article contains supporting information.

## Conflict of interest

A. C. P.-O., A. N. Q., L. R. A., S. E. H., J. A. M., and R. L. L. are coinventors of US Provisional Patent 62/776,799.
