# Supplementary material for: Recombinant antibodies recognize conformation-dependent epitopes of the leucine zipper of misfolding-prone myocilin
Source: J Biol Chem. 2021 Aug 9;297(3):101067. doi: 10.1016/j.jbc.2021.101067 (PMC8408531; doi:10.1016/j.jbc.2021.101067)
Supplement: Figures S1–S7 [file mmc1.pdf]

Recombinant antibodies recognize conformation-dependent epitopes of the leucine zipper of misfolding-prone myocilin

Athéna C. Patterson-Orazem[1^], Ahlam Qerqez[2^], Laura R. Azouz[2^], Minh Thu Ma[1^], Shannon E. Hill[1], Yemo Ku[1], Lisa A. Schildmeyer[1], Jennifer A. Maynard[2,3*], Raquel L. Lieberman[1*]

[1]School of Chemistry & Biochemistry, Georgia Institute of Technology, Atlanta, GA 30332
[2]Department of Chemical Engineering, and [3]Department of Molecular Biosciences University of Texas at Austin, Austin, TX, 78712

[1]School of Chemistry & Biochemistry, Georgia Institute of Technology, Atlanta, GA 30332
[2]Department of Chemical Engineering, and [3]Department of Molecular Biosciences University of Texas at Austin, Austin, TX, 78712

[^]These authors contributed equally
*Co-corresponding authors: raquel.lieberman@chemistry.gatech.edu, maynard@che.utexas.edu

Supplemental Information:

Figure S1. Evaluation of murine immune responses to human and mouse CCLZ.

Figure S2: IgG purification and epitope mapping by dot blot against clarified recombinant *E. coli* lysate.

Figure S3: Myocilin binding by 2A4, 1G12, and 2G9.

Figure S4: Comparison of 1G12, 2A4 and 2H2 IgGs.

Figure S5. Library design and binding specificity of 2H2.

Figure S6: Uncropped blots for 2H2 corresponding to data shown in Fig. 6.

Figure S7: Steady state dissociation constants of 2A4 and 2H2.

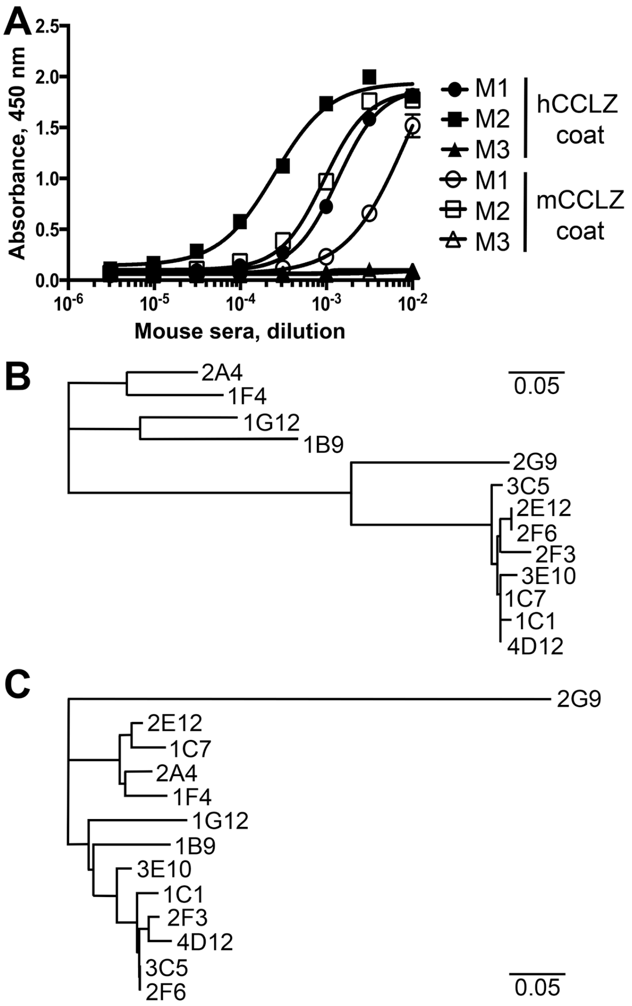

Figure S1. Evaluation of murine immune responses to human and mouse CCLZ. ***A,*** ELISA to measure mouse sera titers after boosting. Multiple sequence alignment of ***B,*** heavy chain and ***C,*** light chain variable region sequences. Panel prepared with Geneious version 8.0.

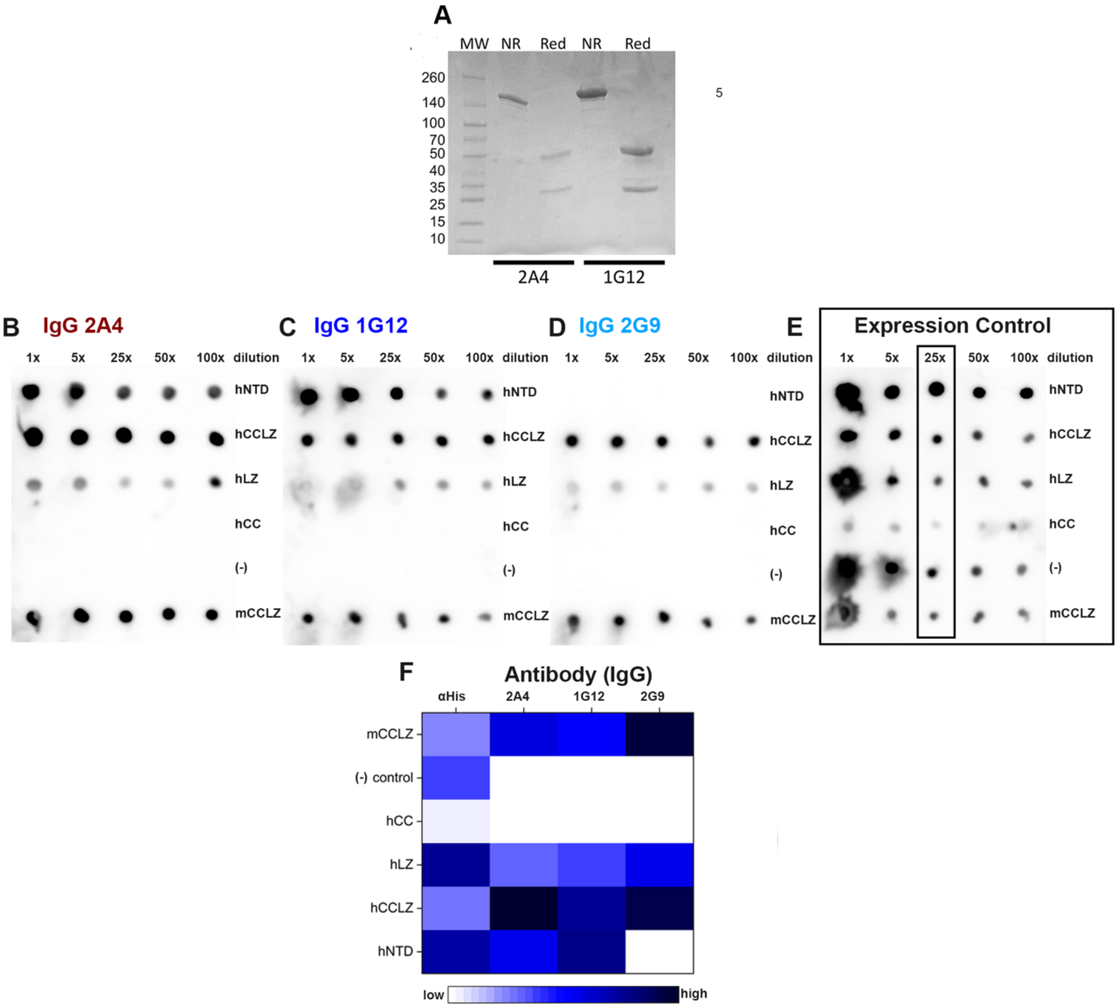

Figure S2: IgG purification and epitope mapping by dot blot against clarified recombinant *E. coli* lysate. ***A***, SDS-PAGE gel of protein A purified 2A4 and 1G12, shown under reduced (Red) and non-reducing (NR) conditions. ***B-E***. Representative dot blots from epitope mapping LZ-targeted IgGs. The spot intensity of the 25x cell lysate dilution (highlighted in E) was determined using Image-J. ***F***. Heat map representation of results (same as Fig. 4A). The average intensity from duplicated independent experiments was used in the heat map function Origin Pro. Increased intensity corresponding to stronger antibody binding (darker spots) in the dot blots translates to darker blue squares in the heat map.

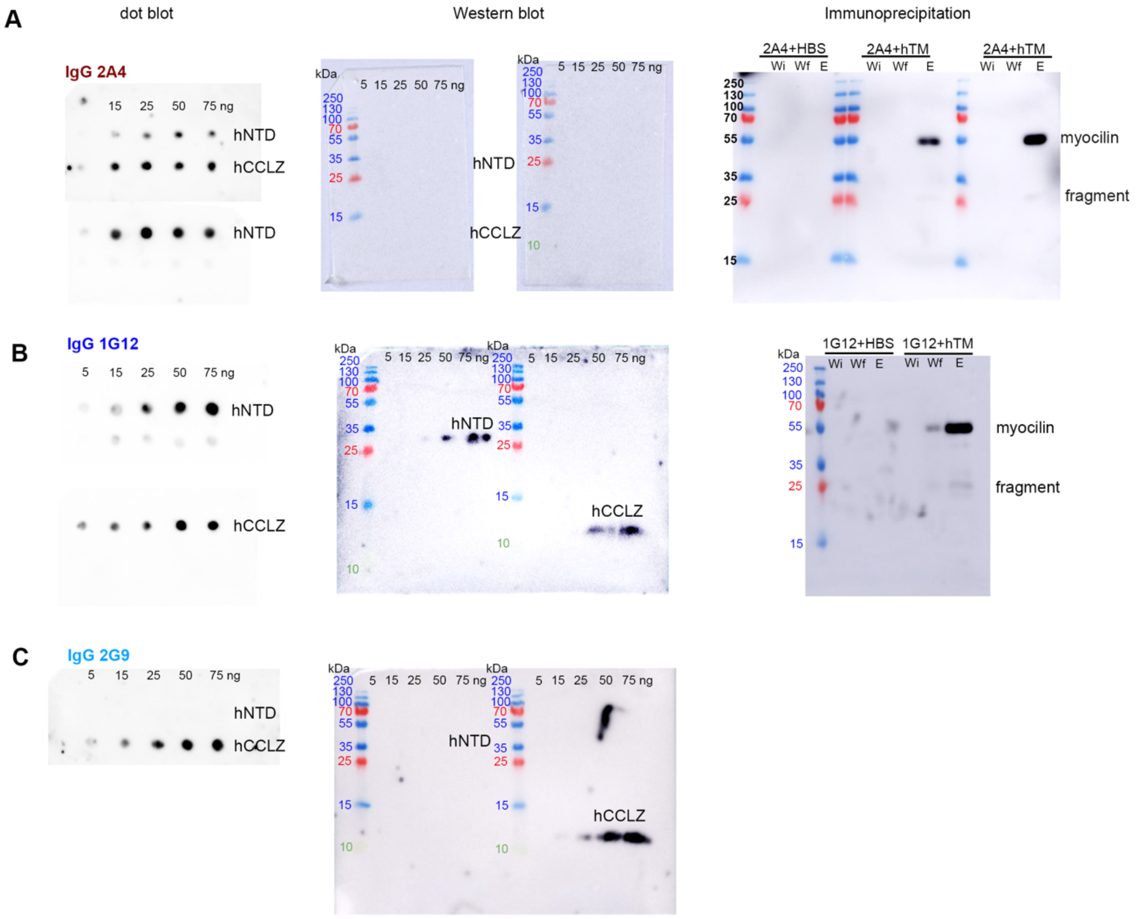

Figure S3: Myocilin binding by 2A4, 1G12, and 2G9. ***A-C,*** Full dot blot, Western blot, and immunoprecipitation blots corresponding to data shown in Fig. 5. For immunoprecipitation of myocilin from human TM (hTM) cell media by custom IgGs results from a media-free negative control (using HBS (50mM Hepes pH 7.5, 200mM NaCl, 10% glycerol), left) and immunoprecipitation experiments (right) are presented. Wi, initial wash step, Wf, final wash step, E, elution step. Arrows indicate full-length myocilin (~55 kDa), and a truncated N-terminal myocilin fragment (~25 kDa).

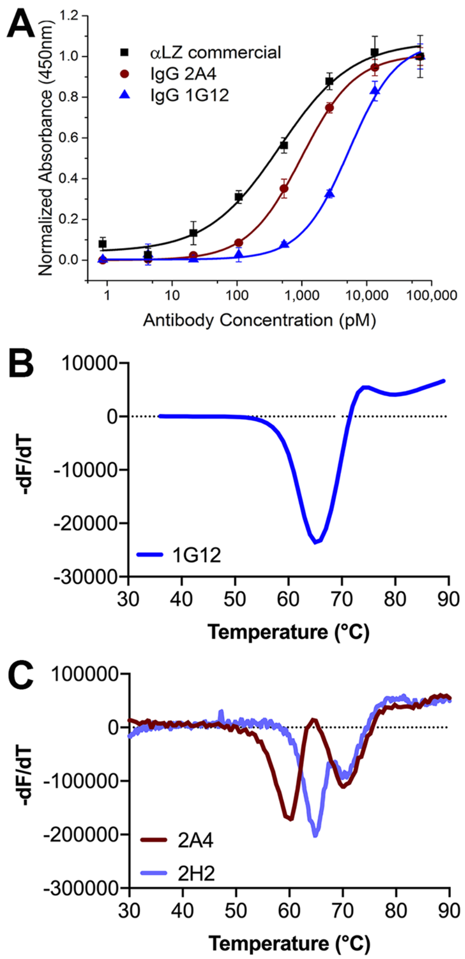

Figure S4: Comparison of 1G12, 2A4 and 2H2 IgGs. ***A,*** ELISA to compare binding affinity towards hNTD of new IgGs with commercial antibodies targeting the human LZ (R&D Systems MAB3446). ***B**, **C*** Thermal stability of the 1G12, 2A4, and 2H2 as SEC-purified human IgG1 proteins measured by differential scanning fluorimetry. Differential plots illustrating the melt transitions as minima for IgG ***B,*** 1G12 (blue) and ***C,*** 2A4 (red) and 2H2 (lavender); average of triplicate measurements shown. Experiment performed at least twice with triplicate technical replicates.

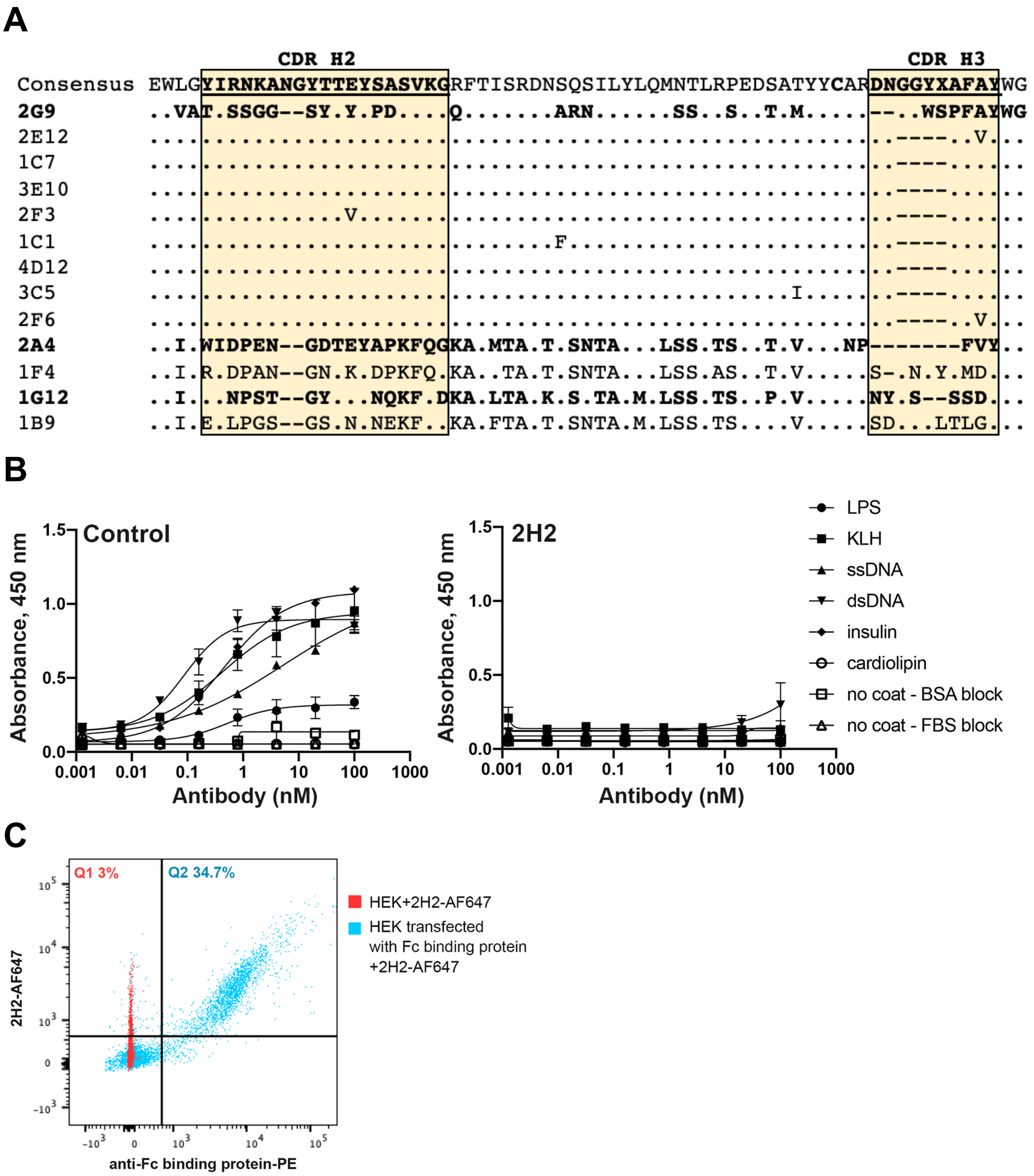

Figure S5: Library design and binding specificity of 2H2. **A,** Sequence alignment of CDR H2 and H3 regions used to design the 2A4 library. A dot indicates the same residue as in the consensus sequence, while a dash indicates a gap in the sequence due to a shorter loop. **B,** ELISAs with a panel of diverse coat antigens to assess polyspecific binding. **C,** 2H2 staining of control HEK293 cells, as measured by flow cytometry. HEK293 cells expressing a high-affinity Fc binding protein (blue population) or untransfected cells (red) were stained with 250 nM Alexa-647-labelled 2H2 and a PE-labelled antibody against the Fc binding protein for one hour on ice. 2H2 stained 3% of untransfected cells versus 35% of transfected cells.

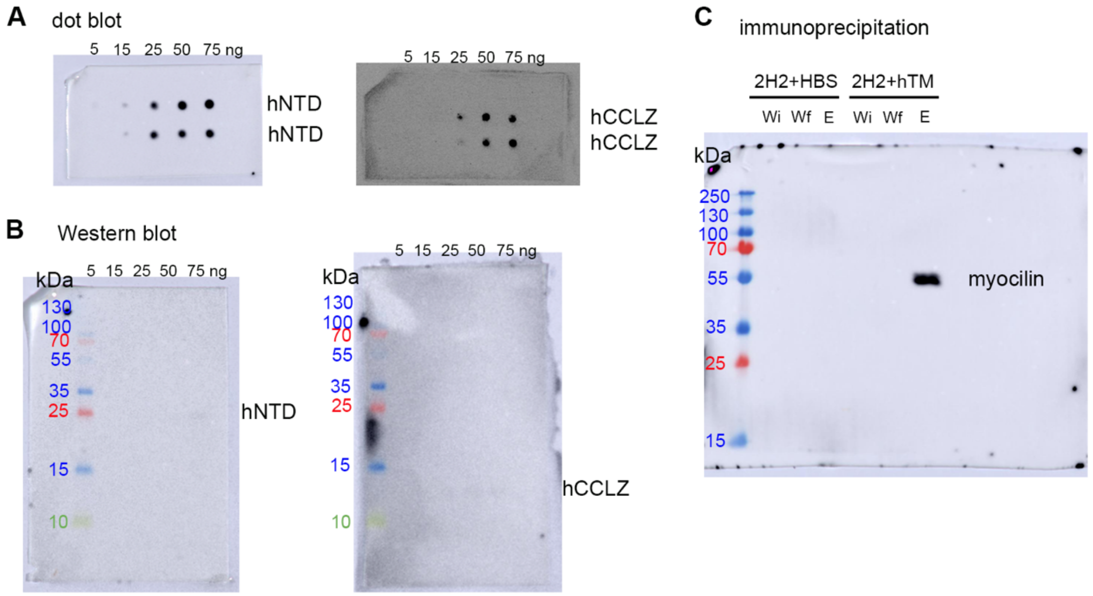

Figure S6: Uncropped blots for 2H2 corresponding to data shown in Fig. 6. A. 2H2 was assessed in dot blots against nanogram quantities of folded hNTD and hCCLZ. Two analytical replicates were performed for each blot. B. 2H2 was assessed in western blots against nanogram quantities of denatured hNTD and hCCLZ. C. Western blot detection of myocilin obtained through immunoprecipitation with 2H2. 2H2 was incubated with either HBS (50mM Hepes pH 7.5, 200mM NaCl, 10% glycerol) or spent hTM media. Protein A/G resin was washed at least 5 times prior to elution. Samples from the first wash (Wi), final wash (Wf), and elution (E) were selected for analysis.

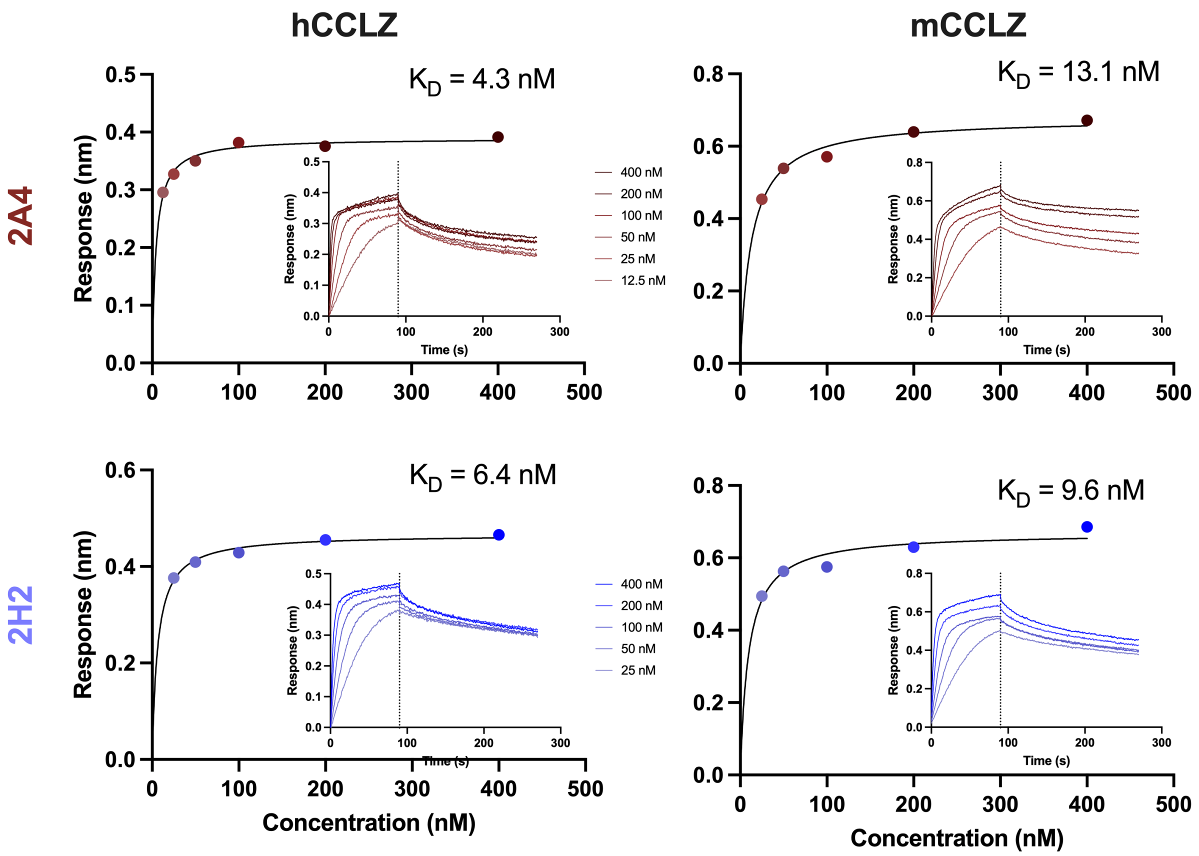

Figure S7: Steady state dissociation constants of 2A4 and 2H2. Binding to hCCLZ and mCCLZ was measured by biolayer interferometry. K_D was assessed by steady state analysis of the association stage shown; 95% confidence intervals in **Table 3**.
